# SILAC-Based Quantitative Proteomic Analysis of Diffuse Large B-Cell Lymphoma Patients

**DOI:** 10.1155/2015/841769

**Published:** 2015-04-28

**Authors:** Ulla Rüetschi, Martin Stenson, Sverker Hasselblom, Herman Nilsson-Ehle, Ulrika Hansson, Henrik Fagman, Per-Ola Andersson

**Affiliations:** ^1^Department of Clinical Chemistry and Transfusion Medicine, Sahlgrenska University Hospital, 413 45 Göteborg, Sweden; ^2^Sahlgrenska Academy, University of Gothenburg, 413 45 Göteborg, Sweden; ^3^Section of Hematology, Kungälv Hospital, 442 83 Kungälv, Sweden; ^4^Section of Hematology and Coagulation, Sahlgrenska University Hospital, 413 45 Göteborg, Sweden; ^5^Department of Pathology and Cytology, Sahlgrenska University Hospital, 413 45 Göteborg, Sweden; ^6^Unit of Hematology, Department of Medicine, Södra Älvsborg Hospital, 504 55 Borås, Sweden

## Abstract

Diffuse large B-cell lymphoma (DLBCL), the most common lymphoma, is a heterogeneous disease where the outcome for patients with early relapse or refractory disease is very poor, even in the era of immunochemotherapy. In order to describe possible differences in global protein expression and network patterns, we performed a SILAC-based shotgun (LC-MS/MS) quantitative proteomic analysis in fresh-frozen tumor tissue from two groups of DLBCL patients with totally different clinical outcome: (i) early relapsed or refractory and (ii) long-term progression-free patients. We could identify over 3,500 proteins; more than 1,300 were quantified in all patients and 87 were significantly differentially expressed. By functional annotation analysis on the 66 proteins overexpressed in the progression-free patient group, we found an enrichment of proteins involved in the regulation and organization of the actin cytoskeleton. Also, five proteins from actin cytoskeleton regulation, applied in a supervised regression analysis, could discriminate the two patient groups. In conclusion, SILAC-based shotgun quantitative proteomic analysis appears to be a powerful tool to explore the proteome in DLBCL tumor tissue. Also, as progression-free patients had a higher expression of proteins involved in the actin cytoskeleton protein network, such a pattern indicates a functional role in the sustained response to immunochemotherapy.

## 1. Introduction

The outcome for diffuse large B-cell lymphoma (DLBCL) patients with early relapse or with refractory disease, even in the era of immunochemotherapy, is dismal and very few are alive after 2 years. Today, the only prognostic tool used in clinical practice to risk-stratify DLBCL patients is the International Prognostic Index (IPI), based on clinical variables at diagnosis (age, performance status, disease stage, extranodal disease, and lactate dehydrogenase in serum) [1] which also has been found to be valid in the immunochemotherapy era, where 3-year progression-free survival ranges from 87% in low-risk patients to 55% in high-risk patients [2]. However, IPI has limitations, and the index seems to be less useful in the identification of the individual high-risk patient [3]. Thus, there is a need for reliable biological markers that can identify these high-risk patients. More than a decade ago, based on the results from global gene expression profiling, two subtypes of DLBCL with different outcomes were described: germinal center B-like (GCB) and activated B-like (ABC) [4], the former with significantly better survival. Furthermore, a simplified gene expression analysis including only 6 genes (*LMO2, BCL6, FN1, CCND2, SCYA3*,and* BCL2*) could also discriminate between these subtypes [5, 6]. Yet, as these methods are so far not standardized, attempts have been made to translate the results from the gene expression analyses to clinically applicable immunohistochemical staining methods. The most important study showed that it was possible to subclassify DLBCL into GCB or non-GCB type using only 3 proteins (CD10, bcl-6, and MUM-1) [7], but the clinical value of these findings has been contradictory [8–10]. In addition, there are many studies pointing out that the mRNA levels do not necessarily reflect the protein expression of cells and tissues, for example, due to posttranscriptional regulation, as well as differences in mRNA and protein turnover rates. Also, the reproducibility of immunohistochemical staining for the most important biomarkers in DLBCL is limited [11, 12]. Instead, another way to search for potential protein biomarkers in DLBCL could be by a proteomic analysis. In recent years, different proteomic approaches have been applied, mostly on cell lines or in animal models, to investigate the proteome in DLBCL tumor material [13–19]. So far, none of the studies have addressed the prognostic challenge in DLBCL, that is, identification of high-risk patients. However, it was recently shown that a SILAC-based quantitative proteomic analysis of different DLBCL cell lines could differentiate between the GCB and ABC subtypes [14]. Even if stable isotope labeling with amino acids in cell culture (SILAC) is a precise method of quantitative proteomics it has been restricted to cell lines that could be metabolically labeled [20]. Recently, this technique has however been improved by using a mix of multiple SILAC-labeled cell lines as an internal standard, achieving a much higher protein quantification precision and also enabling a comparison of proteins not only in cell lines but also in tumor tissue [21]. The aim of our study was to use quantitative shotgun LC-MS/MS proteomic analysis with the SILAC-based technique in fresh-frozen tumor tissue in two groups of DLBCL patients who have been treated with modern immunochemotherapy with totally different clinical outcome, that is, (i) early relapse/refractory patients and (ii) long-term progression-free patients, in order to explore possible alterations in global protein expression and protein network patterns.

## 2. Material and Methods

### 2.1. Selection and Preparation of Tissue Samples

We identified all adult patients with de novo DLBCL diagnosed between January 2004 and December 2008 at the Section of Hematology of Sahlgrenska University Hospital and treated with curative intent immunochemotherapy regimens (i.e., R-CHOP: the monoclonal CD20-antibody rituximab plus cyclophosphamide, doxorubicin, vincristine, and prednisone). We obtained clinical information from the patient casebook including treatment and progression-free and overall survival. Then, we determined two subgroups on the basis of response to initial treatment: (i) patients with primary refractory disease or relapse within 1 year after completion of treatment and (ii) patients considered cured, that is, progression-free with a follow-up of at least 5 years. For the proteomic analysis, five patients from each subgroup were selected based on the availability of freshly frozen pretreatment tumor tissue samples. To avoid obvious morphologic differences between the two groups, the pathologists carefully examined the tumor tissue samples and only samples with evenly distributed blasts without signs of necrosis or abundant visual stroma. In addition, the pathologists had no clinical data for the individual patient when analyzing the tissue sample. Clinical characteristics are described in Supplemental Table 1 (see Supplementary Material available online at http://dx.doi.org/10.1155/2015/841769). Ethical approval for the study was obtained from the Regional Ethics Review Board, Göteborg.

### 2.2. Cell Culture and Preparation of a SILAC Reference Mix

To enable quantification of a broader number of proteins, cell lines established from four human diffuse large B-cell lymphoma patients (Karpas 422, WSU-NHL, SU-DHL5, and SU-DHL8) and from one EBV-negative Burkitt's lymphoma cell line (DG-75) were metabolically labeled with stable isotopes. Cells were cultured for at least six cell doublings in SILAC-RPMI Flex medium (Invitrogen) supplemented with 2 g/L glucose, 2 mM L-glutamine, 100 U/mL penicillin, 100 U/mL streptomycin, 0.1 mg/mL ^13^C_6_-lysine, 0.1 mg/mL ^13^C_6_-arginine, and either 10% (WSU-NHL and DG-75) or 20% (Karpas 422, SU-DHL5 and SU-DHL8) dialyzed fetal calf serum. Full incorporation of labeled amino acids was assessed by mass spectrometry. Fully labeled cells were harvested, washed in PBS, and counted; cells were pelleted by centrifugation in portions of 50 million cells and frozen at −80°C. Fifty million cells from each cell line were thawed and lysed in 500 *μ*L SDS (4% sodium dodecyl sulfate) lysis buffer, 10 mM dithiothreitol (DTT), and 0.1 M Tris-Cl pH 7.6, heated at 95°C for 5 minutes, sonicated for 20–40 seconds, and centrifuged at 16,000 g for 10 minutes to remove cell debris. The protein concentration was determined in the supernatants with the Pierce 660 nm protein assay supplemented with the Ionic Detergent Compatibility Reagent (Thermo Scientific). Equal amounts from each of the five cell extracts were mixed to produce a SILAC reference mix. The mix was aliquoted and stored at −80°C.

### 2.3. Sample Preparation

From each OCT- (optimal cutting temperature-) embedded DLBCL tumor tissue sample, ten cryosections of 100 *μ*m were cut and transferred to polypropylene tubes for protein extraction. Tissue sections were washed twice with 500 *μ*L of PBS (10 mM phosphate, 150 mM NaCl pH 7.2) to remove residual OCT and homogenized in 500 *μ*L SDS lysis buffer (10 mM DTT, 4% SDS, and 0.1 M Tris-Cl pH7.6). Cellular debris was removed by centrifugation at 16,000 g for 10 minutes at +4°C and the protein concentration was determined in the supernatant using the Pierce 660 nm protein assay supplemented with the Ionic Detergent Compatibility Reagent. Protein extracts were stored at −80°C pending analysis.

Prior to protein fractionation, an aliquot corresponding to 30 *μ*g of protein from each tissue sample was mixed with an aliquot corresponding to 30 *μ*g of protein from the SILAC reference mix. Proteins were separated on NuPAGE 4–12% Bis-Tris gels (Life Technologies), gels were fixed in methanol/acetic acid, and proteins were visualized by colloidal coomassie staining (Life Technologies), all following the recommendations of the manufacturer. Gel lanes were divided into 15 equally sized pieces and subjected to trypsin in-gel digestion. The slices were washed in water : acetonitril (1 : 1), dehydrated in acetonitril, and reduced by addition of 10 mM dithiothreitol in 50 mM ammonium bicarbonate (+56°C for 45 min), followed by alkylation with 55 mM iodoacetamide in 50 mM ammonium bicarbonate (30 min at room temperature in the dark). Sequencing-grade modified trypsin (Promega Corp, Madison, WI, USA) dissolved in 50 mM ammonium bicarbonate was added and the digestion was performed overnight at +37°C. All steps of the in-gel digestion were automated and performed using C-18 microfilter 96-well plates (MFNSC18 from Glygen Corp., Columbia, MD, USA) on a Beckman Coulter BioMek 2000 workstation. In a final step, peptides were bound to the C18 resin of the microfilter plate, washed with 0.2% formic acid, and eluted in two times 50 *μ*L of 60% acetonitrile and 0.1% formic acid. In parallel, protein mixtures were also processed by the filter aided sample preparation (FASP) procedure [22]. An aliquot corresponding to 200 *μ*g of protein from each tissue sample was mixed with an aliquot corresponding to 200 *μ*g of protein from the SILAC reference mix and the mixture was suspended in a total volume of 500 *μ*L of SDS lysis buffer (4% SDS, 10 mM DTT, and 0.1 M Tris-Cl pH 7.6). Proteins were reduced, carbamidomethylated, and trypsin-digested following the procedure described by Geiger et al. [23]. Peptides were eluted from the FASP filter devices in two times 40 *μ*L of 40 mM ammonium bicarbonate. The resulting peptides were fractionated by strong anion exchange separation using the Top Tip strong anion exchanger (TT2PSA, Glygen), essentially following the procedure described by Wisniewski et al. Peptides were finally desalted on C18 spin columns (Macro Spin C18 Columns from Nest Group). All peptide mixtures were evaporated to dryness and stored at −80°C until analysis.

### 2.4. Liquid Chromatography-Tandem Mass Spectrometry (LC-MS/MS) Analysis

Samples were redissolved in 20 *μ*L 0.1% formic acid and analyzed by online nanoflow liquid chromatography (LC) (Ettan MDLC, GE Healthcare) coupled to nanoelectrospray ionization (nano-ESI) mass spectrometry (MS) on a LTQ-FT Ultra (Thermo Fisher Scientific) instrument. Peptide mixtures were separated on a 150 × 0.075 mm fused-silica reversed-phase column (Zorbax 300SB-C18, Agilent Technologies) using 240 min (FASP samples) or 120 min (1D SDS-PAGE samples) gradients at a flow rate of 200–300 nL/min. The mass spectrometer was operated in a data-dependent mode to automatically switch between MS and MS/MS acquisition. Survey MS spectra (from* m*/*z *350 to 1500) were acquired in the FT-ICR at a resolution of 50,000, and up to the 10 most intense ions in each FT scan were fragmented and analyzed in the linear ion trap (LTQ).

### 2.5. Protein Identification and Quantification

Raw data, containing full-scan spectra acquired in profile mode and centroid MS/MS spectra, from the analysis of tryptic peptides, were merged and processed using the MaxQuant software version 1.2.0.18 [24]. Default settings were used for feature extraction and first search for recalibration was performed against the human first search database provided with the software. The Andromeda search engine [25] integrated into the MaxQuant package was used for peptide identification and searches were performed against the human subsection of the UniProtKB database. Parameters for identification and quantification were set as follows: variable modification: oxidation of methionine and acetylation of the N-terminal; fixed modification: carbamidomethylation of cysteine, MS/MS tolerance 0.5 Da; peptide and protein false discovery rate (FDR) was set to 0.01 and for SILAC labeled samples the heavy label was set to arg6 and lys6. SILAC protein ratios are determined as the median of all peptide ratios assigned to the protein. For quantification a minimum peptide ratio count of two was set for each protein. To ensure that the Log2 values of the normalized protein H/L ratios followed a normal distribution and were centered to zero; histograms were plotted. A two sample *t*-test was performed to determine significant differences in protein ratios between the groups, Perseus module (version 1.2.0.17) available in the MaxQuant environment.

### 2.6. Validation by Western Blotting

Expression levels of selected proteins were validated by immunoblot analysis of tumor protein extracts from all patients. Equal amounts (10 or 30 *μ*g depending on primary antibody used) of protein were separated on NuPAGE 4–12% Bis-Tris gels in 1xMES buffer (Life Technologies), transferred to nitrocellulose membranes (Hybond ECL, GE Healthcare Life Sciences), and incubated overnight at +4°C with the primary antibody. For signal detection, membranes were incubated with HRP-conjugated secondary antibody (anti-mouse or anti-rabbit) developed with the Super Signal West dura reagent (Pierce) and the signal was recorded by the LAS-3000 luminescent image analyzer (Fujifilm). Primary antibodies used were anti-moesin (ab3196), anti-CAP1 (ab133655), anti-annexin VI (ab52221), and anti-beta II tubulin (ab103667) from AbCam (Cambridge, UK) and secondary antibodies were anti-mouse IgG-HRP (W4021) or anti-rabbit IgG-HRP (W4011) from Promega (Wisconsin, USA). The SILAC reference mix was used as a control.

### 2.7. Multivariate Statistical Analysis by Principal Component Analysis (PCA) and Partial Least-Squares Discriminant Analysis (PLS-DA)

In order to identify potential outliers among the analyzed samples, a PCA analysis was performed using the Log2-transformed H/L ratios for the 1,305 proteins for which a quantitative value was determined in all of the 10 patient samples and the SILAC reference cell line mix. Variables were standard normal variate (SNV) normalized and used as input in a PCA using the SIMCA software (version 13.0.2.0, Umetrics, Sweden). The residual standard deviation, DModX (residual distance, root mean square), was calculated to identify deviations between the data and the principal component model. For selected proteins, a supervised partial least-squares regression analysis was performed using the PLS-DA module of the SIMCA software and the input variables were the H/L ratios determined for the proteins.

### 2.8. Protein Network and Functional Analysis

The differentially expressed proteins in our study were analyzed using the DAVID (Database for Annotation, Visualization and Integrated Discovery) Bioinformatic resources version 6.7 (http://david.abcc.ncifcrf.gov), the PANTHER (Protein ANalysis THrough Evolutionary Relationships) system version 7 (http://www.pantherdb.org), and the STRING (Search Tool for the Retrieval of Interacting Genes/Proteins) version 9.1 (http://string.embl.de/). The list of Uniprot Accession IDs was loaded into the online tool to extract and summarize functional classification. In DAVID, to identify proteins in the most important biological functional groups, the Functional Annotation Clustering tool was used with an EASE threshold of 0.01. Also, to rank the overall importance of proteins, DAVID uses the term “enrichment score”; a higher score for a group indicates that the proteins are involved in more important terms (enrichment score of 1.3 is equivalent to nonlog scale 0.05). PANTHER performs a classification of genes and proteins and provides gene ontology terms, biological processes, molecular function, and pathways. We also used the STRING database for physical and functional interactions among the identified proteins. STRING uses a score to define interaction confidence; all interactions with a confidence score >0.7 (high confidence) were collected.

## 3. Results

### 3.1. Proteomic Analysis: Identification of Differentially Expressed Tumor Proteins Using LC-MS/MS

**Figure 1:**
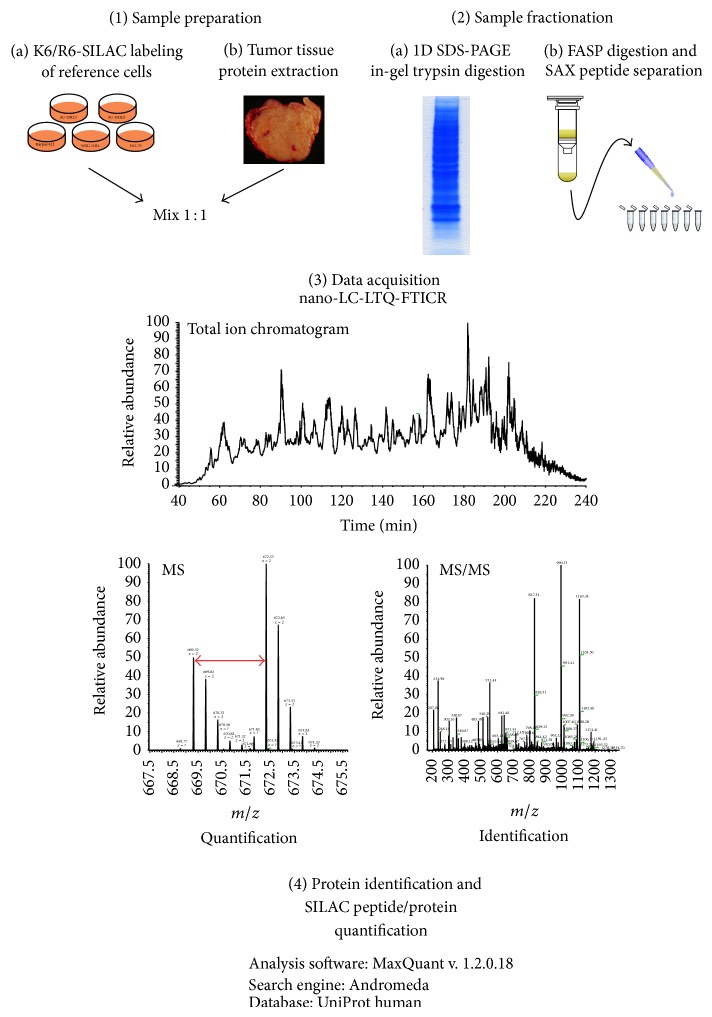
The experimental workflow.

Proteins were extracted from cryosections of frozen tumor biopsies. For the proteomic analysis, five DLBCL patients from each subgroup were selected based on the availability of freshly frozen pretreatment tumor tissue samples. The relative abundance of the extracted proteins was determined by comparison to protein extracts from DLBCL cell lines cultured in medium containing heavy isotope labeled amino acids. To improve coverage and maximize the number of identified/quantified proteins, extracted proteins were processed according to two different workflows. First, proteins were separated by denaturing polyacrylamide gel-electrophoresis and in-gel digested peptides were extracted for further analysis. In parallel, protein extracts were subjected to in-solution digestion according to the FASP protocol followed by peptide fractionation on a strong anion-exchange resin. Proteins were identified by mass spectrometric analysis of tryptic peptides on an LTQ-FTICR hybrid instrument and the relative quantities were calculated based on the ion intensities. The two workflows were run in parallel but the raw data were merged and data processing was performed in one single batch. The experimental workflow is schematically outlined in Figure 1.

In total, 3,588 unique protein groups were identified at 1% FDR, among which B-cell lineage specific markers (e.g., CD20, CD22, CD40, and CD79a) were present as well as proteins involved in B-cell receptor mediated signaling (e.g., mitogen-activated protein kinase 3 (MAPK3), spleen tyrosine kinase (SYK), Bruton's tyrosine kinase (BTK), and protein kinase C (PKC)) (Supplemental Table 2). Recently, a thorough proteome investigation of established DLBCL cell lines has been performed [14], and a comparison of the proteins identified in our study revealed a substantial overlap with 2,572 proteins being identified in both studies; this constitutes 72% of the proteins identified in our study. We successfully quantified 3,027 (84%) of the identified proteins in at least one of the samples. Identification and quantification in all samples were obtained for 1305 proteins and 87 of these proteins were significantly (Student's *t*-test, *P* < 0.05) differentially expressed between the two patient groups (the most functionally relevant proteins are described in Table 1 and all 87 proteins are given in Supplemental Table 3). Sixty-six proteins were overexpressed in the group of progression-free patients; 21 proteins were instead overexpressed in the relapsed/refractory group.

### 3.2. Validation by Western Blotting

**Figure 2:**
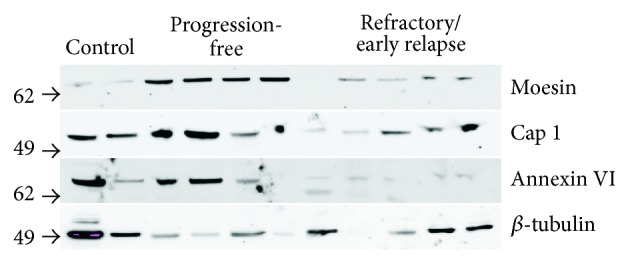
Western blot validation of differences found in the proteomic analysis (for moesin, annexin A6, and CAP1). “Progression-free” represents patients with a follow-up of at least 5 years and “refractory/early relapse” represents patients with primary refractory disease or relapse within 1 year after completion of treatment. The SILAC-reference mix was used as a control and normalization was performed by loading of equal amounts of protein into each lane of the gel. Molecular weight in kDa of the closest migrating band of the SeeBlue marker is indicated in the margin of each panel.

We performed Western blotting to validate the differences seen in the proteomic analysis for moesin, annexin VI, and CAP1, proteins chosen due to their functional characteristics within the actin cytoskeleton network and with sufficient peptide abundance/expression. For these 3 proteins, we could confirm the LC-MS/MS data in the two patient groups (Figure 2).

### 3.3. Multivariate Data Analysis

**Figure 3:**
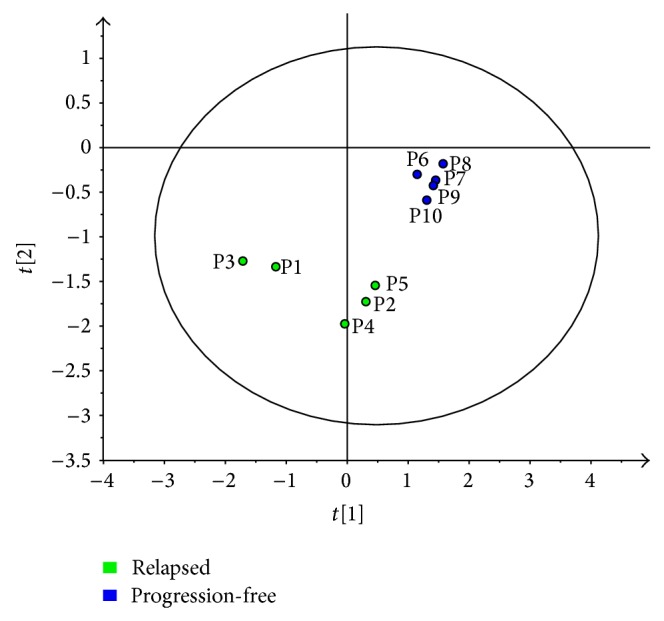
A supervised partial least-squares regression analysis (PLS-DA) including five selected proteins involved in actin cytoskeleton (moesin, CAP1, CAPG, annexin A6, and PDCD4) discriminates the two patient groups. Green dots are patients 1–5 (early relapse/refractory patients) while blue dots are patients 6–10 (progression-free patients).

In order to evaluate the quality of the data, an unsupervised principal component analysis (PCA) was performed. The results showed that all samples were within the 95% confidence interval of the model and no outliers could be detected. Since this type of analysis requires a full dataset without missing data, we used the Log2-transformed H/L ratios for the 1,305 proteins for which a quantitative value was determined in all of the 10 patient samples as well as in the DLBCL cell lines. Next, Log2-transformed H/L ratios for five proteins, that is, moesin, CAP1, actin regulatory protein-G (CAP-G), annexin A6, and programmed cell death protein 4, were used as input variables in a supervised partial-least-squares regression analysis (PLS-DA). The proteins were manually selected based on their involvement in regulation of actin cytoskeleton dynamics in combination with the MS-characteristics, for example, ion intensity and a sufficient number of identified peptides. The group variable was progression-free patients versus patients with refractory disease/early relapse. The PLS-DA model separated the two groups, indicating a discriminating value judged by the R2VY[2] (=0.84) and Q2VY[2] (=0.7) values (Figure 3).

### 3.4. Protein Network and Functional Analysis

**Figure 4:**
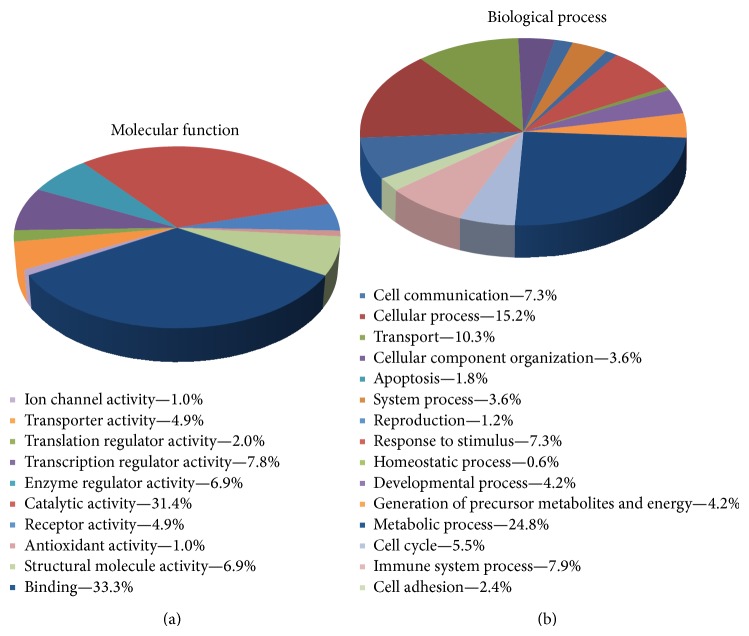
By using the PANTHER database system, the differentially expressed proteins were classified according to molecular function (a) and biological process (b).

**Figure 5:**
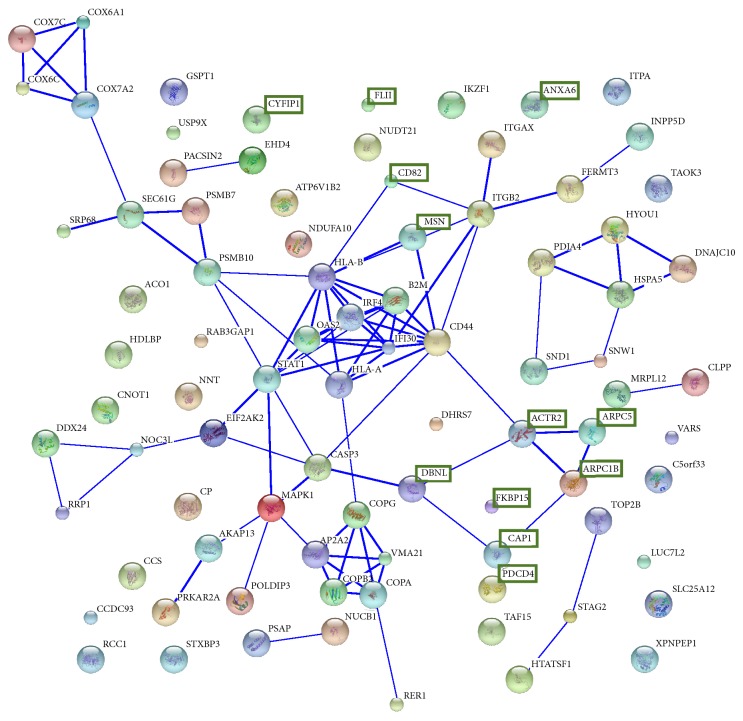
Visualization of network interactions between the 87 differentially expressed proteins by using the STRING database. The figure shows the confidence view and stronger associations are represented by thicker lines. The total number of interactions between the proteins was highly enriched (*P* < 0.00001), as was interactions in the regulation of the actin cytoskeleton network (*P* = 0.0043). Proteins involved in the actin network and actin modulation, which were all overexpressed in the progression-free group, are highlighted with green boxes.

To gain insights into the biological context, all the 87 differentially expressed proteins were subjected to functional characterization using the bioinformatics software DAVID and PANTHER. The DAVID database system, when using an EASE threshold of 0.01, specified 5 functional annotation clusters, ranging from the highest enrichment score: (i) 11 proteins involved in regulation of actin cytoskeleton, (ii) 31 proteins involved in mitochondrial or transmembrane protein networks, (iii) 7 proteins involved in antigen processing, (iv) 22 proteins involved in membrane and intracellular transport, and (v) 21 intraluminal proteins (Table 2). Using PANTHER we classified the proteins according to* molecular function* (the function of the protein by itself or with directly interacting proteins at a biochemical level) and* biological process* (the function of the protein in the context of a larger network of proteins that interact in a process at the level of the cell or organism). For molecular function, the main areas were binding (33.3%), catalytic activity (31.4%), and transcription regulator activity (7.8%) (Figure 4(a)). Regarding analysis of biological processes, proteins involving metabolic process (24.8%), cellular process (15.2%), and transport (10.3%) were the three main groups (Figure 4(b)). Finally, by using STRING database, we could graphically visualize protein-protein interactions and protein networks (Figure 5). Three more tightly connected protein clusters could be suggested: (a) HLA-A/HLA-B/B2M/IRF4/IFI30/CD44, (b) COPA/COPB2/COPG/AP2A2, and (c) ACTR2/ARPC1B/ARPC5/CAP1/DNBL. The total number of interactions between the proteins was highly enriched (*P* < 0.00001), as was interactions in the regulation of the actin cytoskeleton network (*P* = 0.0043).

## 4. Discussion

In this study we could identify, by using SILAC-based LC-MS/MS quantitative proteomic analysis, over 3,500 proteins in fresh-frozen tumor tissue from patients with DLBCL. In addition, we were able to quantify more than 3,000 of the identified proteins in at least one of the samples. The total number of identified and quantified proteins in our study is higher than that reported from the few previous proteomic studies performed so far on tumor tissue from DLBCL patients [16, 17, 26]. However, in these three studies, two-dimensional gel electrophoresis technique for protein separation was used, which partly could explain the lower number of both identified and quantified proteins. Instead, by using gel-free quantitative shotgun LC-MS/MS proteomic analysis with a SILAC-based method on DLBCL cell lines, the sensitivity has recently been found to be considerably higher; the number of identified proteins ranged from 6,569 to 7,756, and quantified proteins ranged from 2,103 to 6,223 [14, 15]. Since cultured cells allow complete metabolic labeling of the whole proteome, analysis of individual tumor cell lines may, at least from a methodological point of view, be more preferable when undertaking quantitative proteomic studies. Yet, analyses of tumor cell lines do not necessarily reflect the tumor biology seen* in vivo* where, for example, the tumor cells also interact with their microenvironment. In that respect, tumor cell lines could perhaps have a more limited role when searching for proteins associated with treatment response, refractoriness, and clinical outcome. Thus, whereas studies on cell lines certainly can be hypothesis generating, it would in that aspect perhaps be more adequate to study human tumor tissue. However, with previous techniques, it has been difficult to perform a relative quantification of the proteome from tissue samples. By using the same approach as in the original super-SILAC paper, the protein identification and quantification sensitivity in our study seems to be in the same range as when breast cancer tissue and breast cancer cell lines were investigated: approximately 5,000 protein groups were then identified and about 4,300 of them were quantified. However, merely identifying a large number of proteins is obviously not enough to pursue possible biomarkers. Yet, as it is well known that a number of alterations in signaling pathways and other cellular processes are involved in the pathogenesis of DLBCL [27, 28], it seems unlikely that single or few biomarkers could mirror the complexity in this disease. Instead, from a biological point of view, it would be more interesting to study patterns or networks of proteins to further understand underlying mechanisms of disease progression and drug resistance. As such, using a SILAC-based LC-MS/MS quantitative proteomic approach appears to be a powerful tool to explore the whole proteome in fresh-frozen tumor tissue from patients with DLBCL. This could broaden the possibilities of using a proteomic platform for finding proteins of differentiating and, hopefully, prognostic significance in this disease.

In our study, about a third of the total number of identified proteins could be quantified in all patients. Among these, we could identify proteins expectedly found in DLBCL tumor tissue, such as B-cell lineage specific markers and proteins involved in the B-cell receptor-signaling pathway. We found that 87 of the quantified proteins were differentially expressed between the two groups of patients, where more proteins were overexpressed in the progression-free patient group (66 versus 21). Among the 66 proteins overexpressed in the progression-free patient group, we found a high proportion of proteins involved in the regulation and organization of the actin cytoskeleton, for example, annexin A6, members from ARP2/3 complex, drebrin-like protein, CAP1, moesin, and WAFL (WASP and FKBP-like) protein. Indeed, by inserting the differentially expressed proteins into the DAVID database, the most enriched annotated cluster was actin-binding proteins or proteins involved in regulation of actin cytoskeleton.Similarly, the STRING database found that interactions in the regulation of the actin cytoskeleton network were highly enriched (*P* = 0.0043). The STRING database analysis also found that the proteins IRF4, B2M, IFI30, HLA-A, HLA-B, and CD44 were another closely connected group of proteins. Most of them are involved in MHC class I signaling and while B2M, IFI30 and HLA-A and HLA-B were all overexpressed in the progression-free group, IRF4 was higher in the relapsed/refractory group. Individually, IRF4 (or MUM-1) has previously been reported to be a negative prognostic marker in DLBCL [7]. CD44, a cell-surface glycoprotein, which was overexpressed in the progression-free group, has been described as negative prognostic marker in DLBCL in the prerituximab period, but immunochemotherapy seems to have diminished this prognostic impact [29]. Yet, as these proteins were previously described in DLBCL, we decided to further investigate proteins involved in the actin network. Accordingly, we then applied five proteins (annexin A6, CAP1, CAP-G, moesin, and programmed cell death protein 4), selected due to their involvement in the regulation of actin cytoskeleton and with a sufficient number of identified peptides, in a supervised regression analysis, which allowed a discrimination of the two patients groups. In addition, by using immunoblotting, we could confirm three of these proteins (annexin A6, CAP1, and moesin) to be overexpressed in the progression-free patient group.

The actin cytoskeleton is essential for many cellular processes, including cell migration, cytokinesis, vesicular trafficking, endocytosis, and morphogenesis. Dysregulation of the actin cytoskeleton is noted in a variety of diseases, such as autoimmune disorders, neurodegenerative diseases, and cancer metastasis [30, 31]. Annexin A6 is a cytosolic protein that binds to negatively charged phospholipids at the plasma membrane. It interacts with a large number of signaling proteins and members of the actin cytoskeleton and is believed to be a key player that links the actin cytoskeleton with the activity of transmembrane proteins [32]. Also, annexin A6 has an inhibitory effect on Ras/MAPK signalling which implies that it could function as a tumor suppressor [33]. The ARP (actin-related protein) 2/3 complex nucleate actin, which leads to the formation and remodeling of cortical actin networks, a configuration also crucial for endocytosis [34]. This complex requires nucleation-promoting factors, mainly belonging to the WASP (Wiskott-Aldrich syndrome protein) family. It has previously been reported that WASPs can act as a suppressor or enhancer for cancer malignancy, depending on the clinical or experimental setting [35]. One of the WASP family members is the protein WAFL involved in regulation of early endocytic transport at the intersection of actin and microtubule dynamics [36]. Another major protein that control actin dynamics is CAP (adenylyl cyclase-associated protein). CAP has two isoforms, CAP1 and CAP2, where CAP1 is ubiquitously expressed in almost all cells [37]. The primary activity for CAP1 is the ability to bind and sequester actin monomers and, depending on cell context, CAP1 can either promote or inhibit cell motility [38]. Moesin is an actin-binding protein belonging to the ERM (ezrin, radixin, and moesin) family. Moesin regulates cell shape changes during cell division and binds and stabilizes microtubules at the cell cortex, thereby mediating the signaling between microtubules and the actin cortex [39]. Interestingly, there is accumulating evidence that the actin cytoskeleton has an important role in B-cell activation and actin remodeling appears to be essential for downregulation of B-cell receptor (BCR) signaling [40]. As BCR signaling is implicated as a pivotal pathway for lymphoma development [28], it is tempting to speculate if an overexpression of actin-modulating proteins could induce a negative effect on the BCR signaling pathway, which could be of functional benefit for patients when treated with immunochemotherapy.

Furthermore, different members of the actin cytoskeleton protein network have been reported to be involved in the mechanisms behind drug resistance against vincristine, a drug included in the standard R-CHOP-regimen used for treatment in DLBCL. A proteomic analysis of a mouse xenograft model of acute B-cell lymphoblastic leukemia has shown that alterations in the actin cytoskeleton are involved in* in vivo* vincristine resistance [41]. Indeed, among 19 proteins displaying altered expression, 11 of them were associated with modulators of the actin cytoskeleton, and most of them were downregulated, for example, moesin, CAP-G, HSP70, and ezrin. Even though the authors stated that the exact mechanism by which a disrupted actin cytoskeleton can induce cellular resistance to antimicrotubule drugs remains to be determined, they concluded that a normal actin cytoskeleton is required for antimicrotubule cellular action of vincristine. Furthermore, Liu et al. most recently performed a proteomic study on fresh-frozen tumor tissue from DLBCL patients divided into two groups: low and high sensitivity to the CHOP chemotherapy regimen [17]. In their study, by using 2-DE and MALDI-TOF/TOF-MS technique, they found that the actin-binding protein ezrin was overexpressed in the high-sensitivity group, as were also the actin-interacting protein pleckstrin and the cytoskeleton-associated annexin V, suggesting that proteins involved in the actin cytoskeleton could be of importance for CHOP chemosensitivity. In addition, it has recently been reported that the monoclonal CD20-antibody rituximab, which is used in combination with CHOP, induces polarization of the CD20 receptor to cap at the B-cell surface which augmented its therapeutic function in NK-cell-mediated antibody-dependent cellular cytotoxicity (ADCC) [42]. Unexpectedly, they found that this action of rituximab was not just only to cluster its CD20 ligand, but also to rearrange several proteins, specifically moesin and ICAM-1, toward the cap. They could then show that an intact microtubule network was required for this CD20 capping.

Taken together, a higher expression of proteins involved in the modulation of the actin cytoskeleton network in DLBCL cells could imply functional importance both for an efficient rituximab-mediated target cell killing by ADCC and for an adequate response to the CHOP regimen, which possibly might explain a better clinical outcome for DLBCL patients when treated with immunochemotherapy. Conversely, the absence of such a pattern could instead indicate resistance or refractoriness to the R-CHOP regimen. Even though intriguing, further studies are needed to confirm and develop these findings.

## 5. Conclusions

In summary, we found that by using SILAC-based shotgun LC-MS/MS quantitative proteomic analysis we could identify and quantify a large number of proteins in fresh-frozen tumor tissue from patients with diffuse large B-cell lymphoma. This could broaden the possibilities of using a proteomic platform for finding proteins of differentiating and, hopefully, prognostic significance in this disease. Our data also indicate that a higher expression of proteins involved in the regulation of the actin cytoskeleton could be of functional importance for a sustained response to immunochemotherapy. As further studies are needed to confirm our findings, we are currently expanding the number of patients, both using fresh-frozen and formalin-fixed paraffin-embedded tissue, to more precisely determine differentiating protein patterns of possible functional and prognostic value in this disease.

## Supplementary Material

Supplemental Table 1: Clinical characteristics of patients in the study. Patients 1–5 had early relapse/refractory disease; patients 6–10 are long term progression-free.
Supplemental Table 2: List of all 3588 proteins that were identified in the SILAC-based proteomic analysis using LC-MS/MS. 3,027 (84%) of the identified proteins were successfully quantified in at least one of the samples. Identification and quantification in all samples were obtained for 1305 proteins.
Supplemental Table 3: All 87 proteins that were significantly (Student's *t*-test, *P* < 0.05) differentially expressed between the two patient groups. 66 proteins were overexpressed in the group of progression-free patients; 21 proteins were instead overexpressed in the relapsed/refractory group.

## Figures and Tables

**Table 1:** The most functionally relevant differentially expressed proteins.

Protein name	ID	Fold change (cur/rel)	*P* value	Function
WAFL	Q5T1M5	6.50	0.021	Regulation of actin and microtubule dynamics
STAT1	P42224	5.87	0.034	Inducer of apoptosis; negative regulation of NF-kappaB signalling
Tetraspanin CD82	P27701	5.15	0.014	Attenuation of plasma membrane-dependent actin organization
EHD4	Q9H223	4.02	0.037	Regulation of endocytic transport
Integrin beta-2; CD18	P05107	3.31	0.048	Transmembrane cell adhesion molecule
Drebrin-like protein	Q9UJU6	3.19	0.019	Actin-binding
ARP2/3 subunit 16	O15511	2.94	0.006	Regulation of actin cytoskeleton
Rac-associated protein-1	Q7L576	2.81	0.041	Regulation of actin cytoskeleton
Saposin C	P07602	2.50	0.020	Antiapoptotic effect via PI3K pathway
SHIP-1	Q92835	2.44	0.038	Involved in B cell receptor signaling pathway
CD11c; integrin alpha	P20702	2.31	0.005	Transmembrane cell adhesion molecule
ARP2/3 subunit 18	O15143	2.15	0.030	Regulation of actin cytoskeleton
Annexin A6	A6NN80	2.08	0.029	Stabilizing cortical actin cytoskeleton
Kindlin-3	Q86UX7	2.03	0.043	Activation and binding partner of integrins
Protein flightless-1 homolog	Q13045	2.01	0.007	Actin binding
CAP1	Q01518	2.00	0.025	Regulation of actin cytoskeleton
MAPK1	P28482	1.92	0.032	Involved in B-cell receptor signaling pathway
ARP2	P61160	1.90	0.035	Regulation of actin cytoskeleton
Syndapin-2	Q9UNF0	1.87	0.014	Linkage of membrane trafficking with the cytoskeleton
Moesin	P26038	1.82	0.027	Actin-binding; stabilizing microtubules at cell cortex
Proteasome MECI-1	P40306	1.77	0.041	Involved in activation of NF-kappa*β* in B cells
JNK/SAPK-inhibitory kinase	Q9H2K8	1.49	0.046	Involved in B-cell receptor signaling pathway via MAPK
Caspase 3	P42574	1.42	0.047	Induction of cell apoptosis
STAG2	Q8N3U4	1.30	0.043	Tumor suppressor
eIF-2A protein kinase	P19525	0.14	0.021	Conserving protein synthesis under environmental stress
CNOT1	A5YKK6	0.34	0.032	Counteracts ER-induced stress apoptosis
NOC3	Q8WTT2	0.35	0.003	Ribosomal; essential for cell division
SKAR	Q9BY77	0.37	0.048	Promotion of cell growth via mTOR and PI3K signaling pathway
eRF3a	P15170	0.39	0.038	Inhibition of apoptosis via survivin
PDCD4	Q53EL6	0.51	0.003	Tumor suppressor via mTOR signaling pathway
MUM-1	Q15306	0.54	0.044	Transcription factor; poor prognostic marker in DLBCL
TAF15	Q92804	0.58	0.001	DNA-binding; induces rapid cell proliferation
RCC1	P18754	0.58	0.015	Chromatin regulator; involved in C-myc transcriptional activation
IKZF1	Q13422	0.61	0.044	Transcription factor; poor prognostic marker in acute lymphoblastic leukemia
SKI protein	Q13573	0.77	0.022	Protooncoprotein

**Table 2:** Annotation clusters according to DAVID using an EASE score of 0.01.

	Name	Number of proteins	*P* value
Annotation cluster 1	Enrichment score 3.07		

	Actin binding	8	0.00012
	Cell projection	7	0.00032
	ARP2/3 protein complex	3	0.00072
	Cytoskeletal protein binding	9	0.0072

Annotation cluster 2	Enrichment score 2.96		

	Mitochondrial inner membrane	4	0.000079
	Hydrogen ion transmembrane transporter activity	6	0.00015
	Inorganic cation transmembrane transporter activity	7	0.0002
	Respiratory chain	5	0.00029
	Monovalent inorganic cation transmembrane transporter activity	6	0.00029
	Membrane-associated complex	4	0.00039
	Cytochrome-c oxidase activity	4	0.00051
	Oxidoreductase activity, acting on heme group of donors, oxygen as acceptor	4	0.00051
	Heme-copper terminal oxidase activity	4	0.00051
	Oxidative phosphorylation	4	0.00051
	Transmembrane protein	11	0.00058
	Electron transfer	4	0.0015
	Generation of precursor metabolites and energy	8	0.0019
	Huntington's disease	7	0.0029
	Oxidoreductase	9	0.0036
	Mitochondrion	11	0.0039

Annotation cluster 3	Enrichment score 2.81		

	Antigen-processing and presentation of peptide antigen	4	0.0005
	MHC class I	3	0.00087
	Heterodimer	5	0.0012
	Antigen processing and presentation of peptide antigen via MHC class I	3	0.004

Annotation cluster 4	Enrichment score 2.78		

	Vesicle coat	5	0.000073
	Retrograde vesicle-mediated transport, Golgi to ER	4	0.00021
	Cytoplasmic membrane-bounded vesicle	12	0.00038
	Membrane-bounded vesicle	12	0.0005
	Membrane coat	5	0.00053
	Golgi vesicle budding	3	0.0014
	Cytoplasmic vesicle	12	0.0014
	Melanosome	5	0.0019
	Pigment granule	5	0.0019
	Membrane budding	3	0.0036
	Golgi membrane	6	0.005
	Golgi apparatus	13	0.0051
	Membrane organization	8	0.0055

Annotation cluster 5	Enrichment score 2.45		

	Nucleolus	12	0.0027
	Intracellular organelle lumen	21	0.003
	Organelle lumen	21	0.004
	Membrane-enclosed lumen	21	0.005
