# Supplementary material for: SILAC-Based Quantitative Proteomic Analysis of Diffuse Large B-Cell Lymphoma Patients
Source: Int J Proteomics. 2015 Apr 28;2015:841769. doi: 10.1155/2015/841769 (PMC4427854; doi:10.1155/2015/841769)
Supplement: Supplementary file 1 — Supplemental Table 1: Clinical characteristics of patients in the study. Patients 1–5 had early relapse/refractory disease; patients 6–10 are long term progression-free. Supplemental Table 2: List of all 3588 proteins that were identified in the SILAC-based proteomic analysis using LC-MS/MS. 3,027 (84%) of the identified proteins were successfully quantified in at least one of the samples. Identification and quantification in all samples were obtained for 1305 proteins. Supplemental Table 3: All 87 proteins that were significantly (Student's t-test, P < 0.05) differentially expressed between the two patient groups. 66 proteins were overexpressed in the group of progression-free patients; 21 proteins were instead overexpressed in the relapsed/refractory group. [file 841769.f1.zip › Suppl Table 3.docx]

| PatNo | Age, y | Sex | Ann Arbor Stage | B-Symptoms  (Yes/No) | S-LDH | Performance (ECOG) | Bulky? | aaIPI | Treatment | Outcome |
| --- | --- | --- | --- | --- | --- | --- | --- | --- | --- | --- |
| 1 | 70 | M | III | Yes | High | 2 | Yes | 3 | R-CHOP-14 | PD |
| 2 | 50 | M | IV | No | Normal | 0 | No | 1 | R-CHOP-21x5 | PD |
| 3 | 60 | F | IV | Yes | High | 3 | Yes | 3 | R-CHOP-14x6 | ER |
| 4 | 85 | M | I | No | Normal | 0 | No | 0 | R-CHOP-21x2 + RT 44Gy | PD |
| 5 | 63 | M | II | No | High | 0 | No | 1 | R-CHOP-21x8 | ER |
| 6 | 55 | F | I | No | High | 0 | No | 1 | R-CHOP-21x8 | PF |
| 7 | 74 | M | III | No | Normal | 0 | No | 1 | R-CHOP-21x6 + R-CHP-21x2 | PF |
| 8 | 58 | F | II | Yes | High | 0 | No | 1 | R-CHOEP-21x8 | PF |
| 9 | 75 | M | II | No | High | 0 | No | 1 | R-CHOP-21x6 | PF |
| 10 | 63 | F | III | No | Normal | 0 | No | 1 | R-CHOP-21x7 + R-COEP-21x1 | PF |

aaIPI: International Prognostic Index (age-adjusted). RT: Radiotherapy. PF: Progression-free with a follow-up of at least 5 years.

ER: Early relapse, i.e. relapse within 1 year after completion of treatment. PD: Progressive disease during treatment.
